# ^68^Ga-PSMA-11 PET/CT in primary staging of prostate carcinoma: preliminary results on differences between black and white South-Africans

**DOI:** 10.1007/s00259-017-3852-8

**Published:** 2017-11-04

**Authors:** Mike Sathekge, Thabo Lengana, Alex Maes, Mariza Vorster, JanRijn Zeevaart, Ismaheel Lawal, Thomas Ebenhan, Christophe Van de Wiele

**Affiliations:** 1 Department of Nuclear medicine, University of Pretoria, Pretoria, South Africa; 2 Department of Nuclear Medicine, University of Pretoria and Steve Biko Academic Hospital, Private Bag X169, Pretoria, 0001 South Africa; 3 Department of Nuclear Medicine, AZ Groeninge, Kortrijk, Belgium; 4 Department of Nuclear Medicine and Pathology, KULAK, Kortrijk, Belgium; 5 Radiochemistry, The South African Nuclear Energy Corporation SOC Ltd (Necsa), Pelindaba, Pretoria, South Africa; 6 Department of Radiology and Nuclear Medicine, University Ghent, Ghent, Belgium

**Keywords:** 68Ga-PSMA, Prostate carcinoma, Racial differences

## Abstract

**Purpose:**

The incidence of prostate cancer is 60% higher and the mortality rate is two- to three-times greater in black versus white men. We report on differences in ^68^Ga-PSMA-11 PET/CT imaging findings in 77 black South-African (BSAs) and 18 white South-African (WSAs) treatment-naïve primary prostate carcinoma (PPC) patients.

**Methods:**

^68^Ga-PSMA-11 PET/CT imaging findings were compared to histological, biochemical and morphological imaging data. Patients were grouped into three Gleason grade groups (GG), GG 1 (scores 3 + 3 and 3 + 4), GG2 (scores 4 + 3 and 4 + 4) and GG3 (scores 9 and 10), and the PSA difference among the groups was determined. Inter-racial difference in SUVmax of the primary tumor as well as its correlation with serum PSA were also determined.

**Results:**

Ninety-three out of 95 PPC where readily identified on ^68^Ga-PSMA-11 PET/CT imaging. Median PPC SUVmax and serum PSA values proved significantly higher (*p* = 0.033 and *p* = 0.003) in GG3 patients (median 16.4 and 180 ng/ml) when compared to GG1 patients (median 9.6 and 25.1 ng/ml) or GG2 patients (median 8.8 and 46.2 ng/ml). SUVmax significantly correlated with serum PSA-values (*r* = 0.377 (*p* = 0.0001)). Age, frequency of lymph node involvement and distant metastases, and GGs (*p* ≥ 0.153) were similar in BSAs and WSAs, both median serum PSA-values as well as SUVmax values proved significantly higher in BSAs when compared to WSAs, respectively, 81.6 ng/ml versus 14.5 ng/ml (*p* = 0.0001) and 11.9 versus 4.38 (*p* = 0.004). Moreover, Gleason-score normalized median SUVmax values proved 2.5 times higher in BSAs when compared to WSAs (*p* = 0.005).

**Conclusion:**

SUVmax values proved significantly related to GG and to be significantly higher in BSAs when compared to WSAs. Also, SUVmax significantly correlated with serum PSA values, which was significantly higher in BSAs when compared with WSAs.

## Introduction

Prostate cancer is the most frequently diagnosed visceral cancer and the second leading cause of cancer death in men [1]*.* Prostate cancer incidence and mortality are significantly higher in black Africans compared with Caucasians [2]. Various factors have been suggested to contribute to the disproportionate advanced disease and the two- to three-times higher mortality from prostate carcinoma in black African men compared with white men. These include access to care, financial barriers or the lack of insurance, comorbidity and socio-economic status (3–5)*.* On the other hand, it has been suggested that racial differences in tumor biology resulting in a faster prostate cancer growth rate and/or earlier transformation from indolent to aggressive prostate carcinoma when compared with Caucasians, likely attributable to differences in dietary, hormonal, or molecular factors, may also contribute significantly to the racial disparity of advanced prostate carcinoma in African black men [3–5]*.*

Prostate-specific membrane antigen (PSMA) is an integral membrane protein, mapped to chromosome 11q14, that is over-expressed by a high number of prostate carcinomas; this expression is further increased in higher grade carcinomas, in metastatic disease and in hormone refractory prostate carcinomas suggesting a role for this antigen in growth and progression and making it an interesting target for prostate carcinoma specific imaging and therapy. After many years of preclinical research on PSMA ligands, the breakthrough was achieved in 2011 with the clinical introduction of Glu-NH-CO-NH-Lys(Ahx)-HBED-CC (^68^Ga-PSMA-11) for PET imaging and 131I–MIP-1095 for radioligand therapy of prostate cancer [6–12].

In this study, we investigated the relationship between ^68^Ga-PSMA-11 imaging findings, Gleason score and PSA serum-levels at initial staging in a South-African population, comprising both white and black men. Our study hypothesis was that more aggressive disease characteristics (higher Gleason score and PSA levels) will be seen in Black South-Africans compared with White South-Africans with treatment-naïve prostate cancer, and that these characteristics will correlate positively with PSMA uptake in the primary tumor and presence of distant metastases on ^68^Ga-PSMA-11 PET/CT.

## Materials and methods

### Patients

Ninety-five patients (mean age: 66 years, range: 43–84 years) with newly diagnosed, treatment naïve prostate carcinoma were prospectively recruited (Jan 2016 to March 2017) for this single-center study, approved by the Institutional Ethics Committee, following written informed consent. There were seventy-seven black South-African men and eighteen white South-African men. In all patients, ^68^Ga-PSMA-11 PET/CT imaging was performed and results obtained compared to histological, biochemical and available imaging data performed as part of the staging. Patients were grouped into three grades based on their Gleason score, respectively group 1 corresponding to mild aggressive lesions (Gleason score 3 + 3 and Gleason score 3 + 4), group 2 (Gleason score 4 + 3 and Gleason score 4 + 4) corresponding to moderately aggressive lesions and group 3 (Gleason scores 9 and 10), corresponding to very aggressive lesions.

## Methods

The PSMA kit was obtained from ABX advanced biochemical compounds (Biomedizinische Forschungsreagenzien GmbH, Radeberg, Germany). Ga-68 was obtained from a Ge-68/Ga-68 generator (iThemba LABS, Somerset West, South Africa). Synthesis of ^68^Ga-68 PSMA-11 was done in-house as previously reported [13]. Radiochemical purity was above 98% in all syntheses administered to patients for imaging.

^68^Ga-PSMA-11 PET/CT imaging: Mid-thigh to vertex ^68^Ga-PSMA-11 PET imaging was acquired in all patients 1 hour following the injection of a bodyweight adjusted dose of 2 MBq/kg [6]. All ^68^Ga-PSMA-11 injections contained 2 nmol PSMA ligand resulting in a median specific radioactivity of 66GBq/μmol [10]. Imaging was acquired on a Biograph 40 Truepoint PET/CT scanner (Siemens Medical Solution, IL, USA). Patient were not subjected to any special preparation prior to imaging. Patients were made to empty their urinary bladder just before imaging was commenced. CT was done without oral or intravenous contrast administration. Its parameters were adjusted for patients’ weight (120KeV, 40-150mAs) with a section width of 5 mm and pitch of 0.8. PET imaging was acquired in 3-D mode at 4 min per bed position after CT imaging. CT data were used for attenuation correction. Image reconstruction was done with ordered subset expectation maximization iterative reconstruction algorithm (four iterations, eight subsets). A Gaussian filter was applied at 5.0 mm FWHM. Frusemide injection was not routinely used during imaging. When a focus of increased tracer uptake could not be clearly identified as a pathologic node as different from urine collection within the ureter, patients were given 500mLs of intravenous normal saline and a limited imaging of the region acquired after voiding.

Acquired ^68^Ga-PSMA-11 PET/CT images were interpreted independently by two board-approved nuclear medicine physicians; disagreement was resolved by consensus. The reconstructed PET/CT images were displayed on a dedicated workstation equipped with syngo software (Siemens Medical Solutions, IL, USA). For calculation of the maximum standardized uptake values of the primary tumor, volumes of interest were drawn using a manually adapted isocontour threshold centered on the corresponding tumor site verified by TRUS biopsy and MRI-imaging. To eliminate the impact of differences in Gleason scores of primary prostate carcinoma in white versus black men included in the study, SUVmax values were normalized for Gleason-score. Normalization was done by dividing the SUVmax of the primary tumor by the Gleason score. Furthermore, uptake higher than background-activity in lymph nodes and tissues, not corresponding to physiologic tracer accumulation, was considered pathologic and compatible with malignancy.

### Statistical analysis

Statistical analysis was performed using SPSS software, version 23.0 (IBM Corp., Armonk, NY). Normalcy of data was assessed using the Kolmogorov-Smirnov test. For comparison of groups, the parametric Student t-test and the non-parametric Mann-Whitney U test and Kruskal-Wallis test were used where appropriate. For correlation analysis, we used the non-parametric Spearman-rank test. For comparison of frequencies, the Chi-square test was used. Linear regression was used to assess those factors significantly contributing to ^68^Ga-PSMA-11 uptake by the primary tumor. The significance level used was *p* ≤ 0.05 (two-tailed).

## Results

### Total group of patients

Median Gleason score of the patient cohort was 7 (range 6–10). Median Gleason grade of the total cohort was 2 (range: 1–3). Median PSA value was 59 ng/ml (range: 0.16–3666 ng/ml). Median SUVmax value of the primary tumor (uncorrected for Gleason score) was 11.2 (range: 2–63.7) (see Table 1).

**Table 1:** Patient Characteristics

Patients (n)	95
-Black South-Africans	77
-White South Africans	18
Age (mean, range)	66 years (43–84 years)
Gleason group 1 (n)	28
Gleason group 2 (n)	28
Gleason group 3 (n)	39
PSA median (range)	59 ng/ml (0.16–3666 ng/ml).

n = number of patients, Gleason group 1 (Gleason score 3 + 3 and Gleason score 3 + 4), Gleason group 2 (Gleason score 4 + 3 and Gleason score 4 + 4) and Gleason group 3 (Gleason scores 9 and 10)

**Fig. 1:**
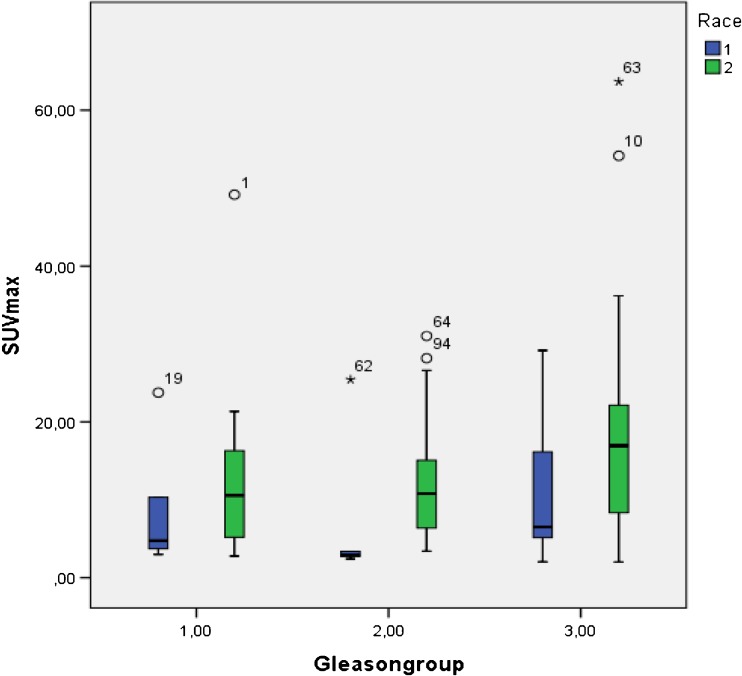
Comparison of SUV max values of primary prostate carcinoma for the different Gleason groups according to race (Blue color corresponds to the results obtained in white men, the green color to results obtained in black men)

**Fig. 2:**
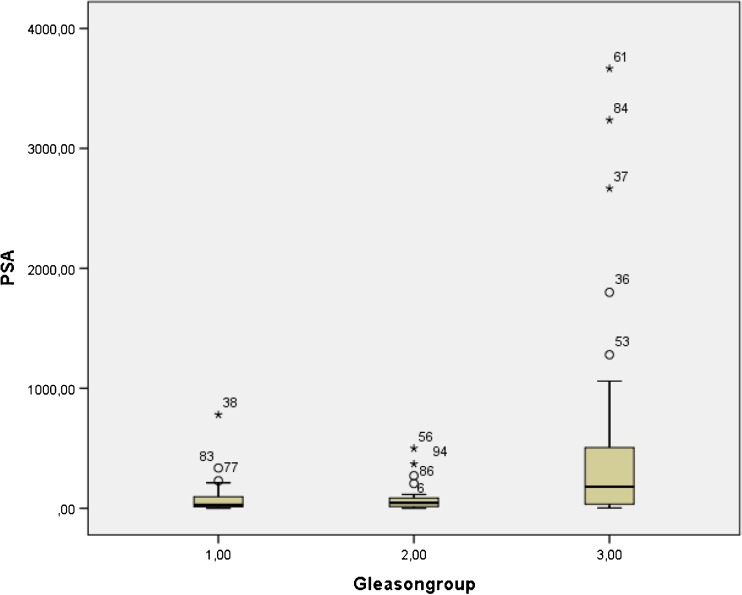
Comparison of PSA values for the different Gleason groups (1 to 3) of the entire cohort

Group 1 (Gleason score 3 + 3 and Gleason score 3 + 4) included 28 patients, group 2 (Gleason score 4 + 3 and Gleason score 4 + 4) also included 28 patients and group 3 (Gleason scores 9 and 10) included 39 patients. With the exception of two patients, all primary tumors showed a higher ^68^Ga-PSMA-11 uptake in their primary tumor when compared with the normal prostate background. SUVmax values proved significantly higher (*p* = 0.033) in group 3 patients (median 16.4 (range (2–63.7)) when compared with patients in group 1 (median 9.6 (range 2.8–49.2) or group 2 (median 8.8 (range: 2.4–31.0) (see Fig. 1). Serum PSA-values values also proved significantly higher (*p* = 0.003) in group 3 patients (median 180 ng/ml (range (2.2–3666 ng/ml)) when compared to group 1 patients (median 25.1 ng/ml (range 1.17–780 ng/ml) or group 2 patients (median 46.2 ng/ml (range: 0.16–499 ng/ml) (see Fig. 2).

**Fig. 3:**
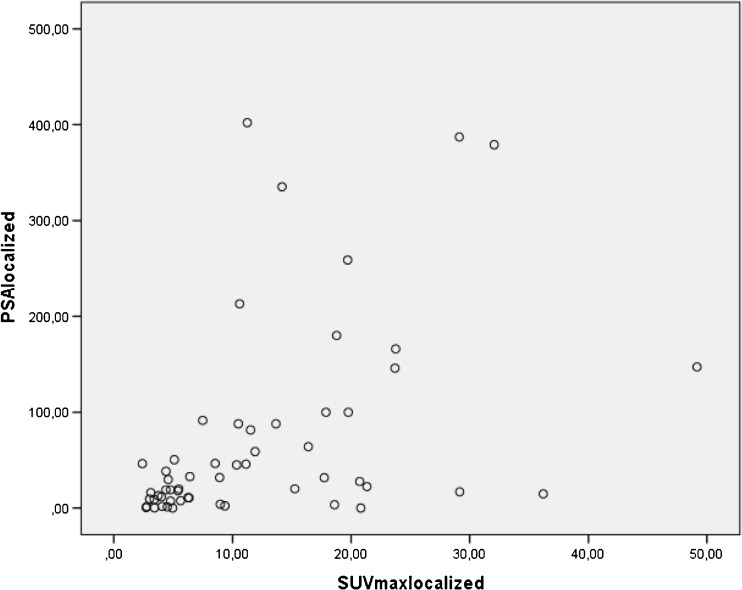
Scatterplot showing the relationship between PSA values and primary tumor related ^68^Ga-PSMA-11 uptake expressed as SUVmax in patients with localized (that is excluding lymph node involvement and/or distant metastases) prostate carcinoma (r = 0.548 (*p* = 0.0001))

Thirty-seven out of ninety-five patients presented with lymph node involvement and/or distant metastases on ^68^Ga-PMSA-11 PET/CT imaging. In these patients, both the SUVmax value of the primary tumor and the serum PSA values were significantly higher when compared with those without lymph node involvement and/or distant metastases, respectively 14.8 (range 2.0–63.7) versus 9.2 (2.0–49.2) for SUVmax values (*p* = 0.043) and 200 (range 3.3–3666) versus 30.1 (range: 0–1280) ng/ml for PSA values (*p* = 0.0001). Also, in the group of patients without lymph node involvement and/or distant metastases, SUVmax values showed a statistically significant moderate correlation with serum PSA-values (*r* = 0.548 (*p* = 0.0001)). This correlation remained significant but becomes a weak correlation (*r* = 0.377 (*p* = 0.0001)) when considering the entire cohort of patients (see Fig. 3).

### Black versus white South-African patients (see Table 2)

**Fig. 4:**
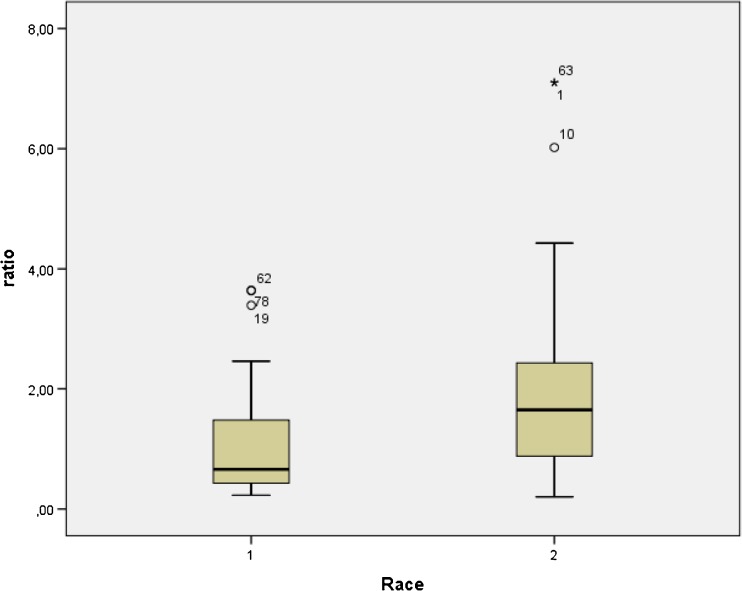
Comparison between the ratio of SUV max values of primary prostate carcinoma to the Gleason score (ratio) versus the race of the patients (1 corresponds to the results obtained in white South-Africans, 2 corresponds to the results obtained in black South-Africans)

**Fig. 5:**
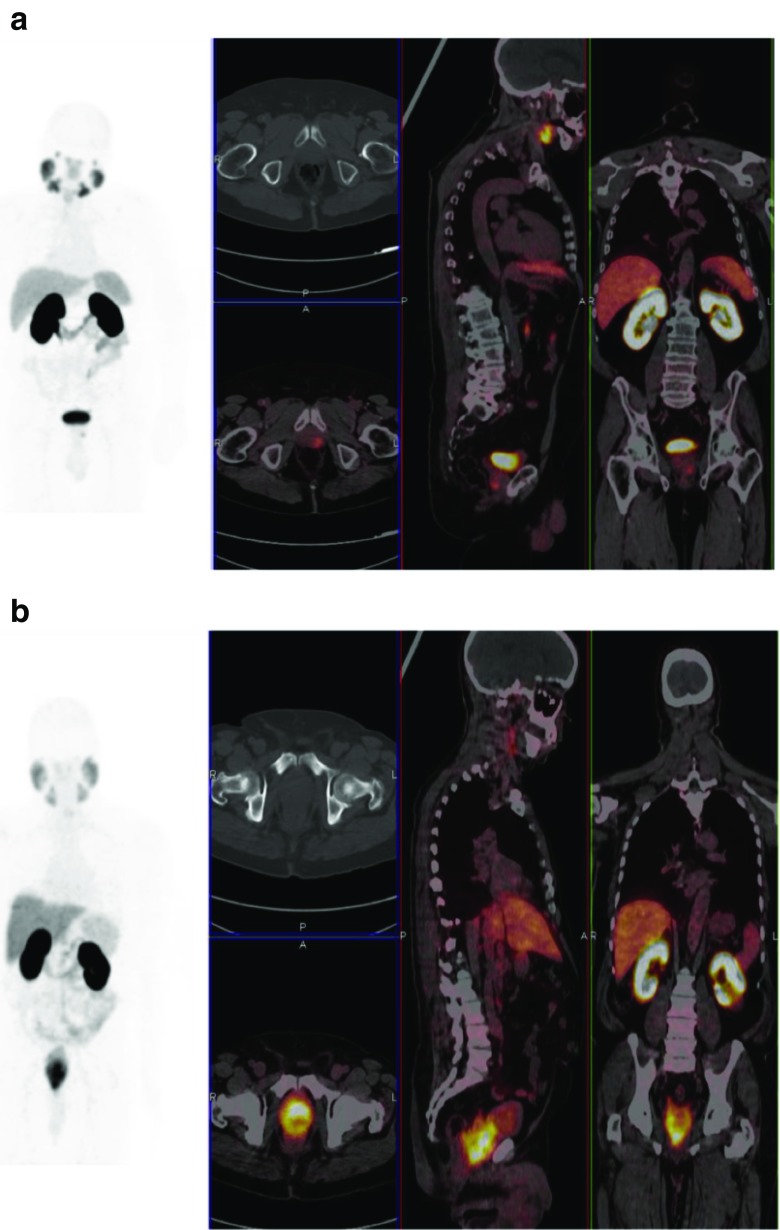
Comparison of SUV max values of primary prostate carcinoma for two patients with the same Gleason scores (4 + 5) but different races (A) corresponds to a 70-year-old white man, maximum-intensity-projection PET, axial, coronal and sagittal fused PET/CT demonstrated SUVmax of 6.3 in the primary prostate cancer with a PSA of 10.5 ng/mL. (B) corresponds to a 69-year-old black man, maximum-intensity-projection PET, axial, coronal and sagittal fused PET/CT demonstrated SUVmax of 29.2 in the primary prostate cancer with a PSA of 38.71 ng/mL

Age proved not significantly different between white (*n* = 18, mean age: 67.6 yrs. (SD: 6.0 yrs) and black South-Africans (mean age = 65.5 (SD8.7) yrs) included in the study (*p* = 0.153). Also, the frequencies of the presence of distant metastases and lymph node involvement proved not significantly different between both groups (respectively, *p* = 0.548 and *p* = 0.292, respectively). Median Gleason scores proved not significantly different between black and white South-African patients **(**respectively, both 7, *p* = 0.964)**.** Conversely, both serum PSA values as well as SUVmax values of the primary tumor proved significantly higher in black South-Africans when compared with white South-Africans, respectively (median) 81.6 ng/ml (range: 0–3660 ng/ml) versus 14.5 ng/ml (range: 0.4–460 ng/ml) (*p* = 0.0001) and 11.9 (range: 2.0–63.7) versus 4.4 (range: 2.03–29.2) (*p* = 0.004) (see Figs. 4 and 5). More importantly, median SUVmax values of the primary tumor normalized for Gleason score proved 2.5 times higher in black men when compared to white men (respectively, median 2.5 (range: 0.20–7.10) versus 0.66 (range: 0.23–3.64), *p* = 0.005) (see Fig. 4). Using linear regression, both Gleason grade (*p* = 0.032) and race (*p* = 0.047) showed significant association with SUVmax values of the primary tumor.

**Table 2:** Differences in Black versus White South-Africans

	Black South-Africans	White South-Africans	p-value
-Nb of patients	77	18	
-Age	65.5 yrs. (sd:8.7 yrs)	67.6 yrs. (sd:6.0 yrs)	0.153(NS)
-SUVmaxPSMAprim	11.9 (range:2.0–63.7)	4.4 (range:2.03–29.2)	0.004*
-SUVmaxPSMA/Gleason score	1.65 (range:0.2–5.1)	0.66 (range:0.23–3.64)	0.005*
-Gleason score	7.0 range6–10)	7 (range 6–9)	0.964
-Gleason group 1(n)	23	5	
-Gleason group 2(n)	22	6	
-Gleason group 3(n)	32	7	
-PSA	81.6 (0.16–3660 ng/ml)	14.5 (0.4–460 ng/ml)	0.00001*
-N-positive	25 (33%)	4 (22%)	0.292(NS)
-M-positive	19 (25%)	4 (22%)	0.548(NS)

Nb = number, n = number of patients, age is given as mean, SD = standard deviation), SUVmaxPSMAprim = SUVmax of primary prostate carcinoma, SUVmaxPSMA/Gleason score = SUVmax of primary prostate cancer corrected for Gleason score, SUVmaxPSMA/Gleason score as well as PSA-values and Gleason score are given as median and range, N = node positive, M = metastasis positive

## Discussion

To our knowledge, this work represents the first attempt at demonstrating the inter-racial differences in tumor biology using^68^Ga-PMSA-11 PET/CT imaging in men with prostate cancer. In the series presented, with the exception of two primary tumors (2% of the patient population studied), all primary tumors were clearly discernible on ^68^Ga-PSMA-11 PET/CT imaging. The two patients in whom the primary tumors were not visualized were white South-Africans. Previous studies in Caucasians have reported on a somewhat lower detection rate with 7.1 to 8.9% of primary prostate carcinoma showing no increase or only a slight increase in ^68^Ga-PSMA-11 accumulation [14, 15]. More specifically, Maurer et al. observed that out of 130 patients, 8.4% of primary prostate carcinoma showed no increase or only a slight increase in ^68^Ga-PSMA-11 accumulation when compared to normal prostate tissue 68Ga-PSMA accumulation [14]. Likewise, Budaüs et al. and more recently Uprimny et al. found a false negative rate for primary prostate carcinoma detection, that is the primary tumor being not distinguishable from the surrounding normal prostate tissue, in respectively 7.1% and 8.9% of patients studied [15, 16]. The more favorable results obtained in our series may in part relate to the preferential inclusion of black South-Africans. As shown in this study, median ^68^Ga-PSMA-11 uptake by primary prostate carcinomas normalized for Gleason score, proved significantly higher in black South-Africans when compared with white South-Africans making them more readily discernible from the normal prostate background. The higher uptake of ^68^Ga-PSMA-11 found in primary prostate carcinoma of black South-Africans when normalized for Gleason core, reflecting PSMA expression and biological activity, may in part explain the more aggressive behavior of prostate carcinoma in black men when compared with white men; respectively, a disproportionate reported advanced disease and a two- to three-times higher mortality from prostate carcinoma in black African men when compared to Caucasians [1–5]. PSMA acts as a glutamate carboxypeptidase on different substrates such as polyglutamated folates and the neuropeptide N-acetyl-l-aspartyl-l-glutamate [17–21]. As shown previously by Lapidus et al., using a N-acetylaspartylglutamate (NAAG) hydrolytic radioenzymatic assay to quantify the enzymatic activity of PSMA in normal, benign prostate hyperplasia (BPH) and prostate cancer tissues from radical prostatectomies, PSMA enzyme activity proved significantly elevated in prostate cancer when compared to normal prostate tissues and BPH [22]. The major constant product of the enzymatic activity of PSMA is glutamate, which was shown to be present in excess amount in prostate carcinoma, even more so in African-American patients when compared to Caucasian-American research subjects, and to favor higher growth rates and aggressive behavior in human prostate carcinoma cell lines [21]. Likewise, prostate carcinoma cells where shown to proliferate directly in response to available folate, made available through PSMA-mediated hydrolysis of polyglutamated folates [19, 20]. PSMA was also shown to facilitate integrin signaling and p21-activated kinase activation, leading to both productive invasion and downregulation of beta1-integrin (a cell-matrix linker), to induce chromosomal instability, to play a VEGF-independent role in neoangiogenesis and to recruit a functionally active complex present in high grade prostate carcinoma patients associated with EGFR phosphorylation [23–26]. The latter receptor, which plays a critical role in prostate cancer signal transduction and progression, was shown to be significantly overexpressed in African Americans when compared to white Americans in a multivariate model after controlling for grade, stage and pretreatment simultaneously [27].

A statistically significant correlation between ^68^Ga-PSMA-11 uptake by primary prostate carcinoma and serum PSA values has been previously reported by Sachpekidis et al. and more recently also by Uprimny et al. [16, 28]. In line with their findings as well as previous studies reporting on elevated immunohistochemical expression and elevated enzymatic activity of PSMA in advanced prostate cancer, we also found a significant correlation between ^68^Ga-PSMA-11 uptake by primary prostate carcinoma and serum PSA [22]. This correlation, which was moderate when only patients with localized disease were evaluated, becomes weak following inclusion of all study cohorts in the evaluation. This weakening of relationship after inclusion of patients with metastatic disease probably reflects the decoupling of SUVmax and serum PSA seen in advanced prostate cancer. A limited number of studies have shown previously that serum PSA is determined by both prostate cancer volume as well as by the percentage of high-grade cancer cells at the time of initial diagnosis [29, 30]. Of interest, both the SUVmax value of the primary tumor and PSA-values proved significantly higher in patients with lymph node involvement and/or distant metastases when compared to those without lymph node involvement and/or distant metastases. The link between elevated PSMA expression by the primary prostate carcinoma and global prostate carcinoma tumor load, as reflected by serum PSA, is currently unclear and has yet to be fully elucidated.

Finally, in the study presented, serum PSA-values in black South-Africans proved significantly higher when compared to white South-Africans, in spite of the absence of a significant difference in the frequency of lymph node and /or lymph node involvement between white and black South-Africans. While this in part reflects the well-documented higher known disease burden found in these patients at initial staging, androgen receptor mutations and polymorphisms regulating PSA production as well as PSA gene polymorphisms between black and white South-Africans may also contribute to this finding [31, 32]. In this regard, a number of studies reported that black men have higher serum prostate-specific antigen values than white men and that among men undergoing radical prostatectomy, black men present with higher serum PSA values, not related to prostate size or pathological cancer-specific characteristics [33, 34].

### Shortcomings of the study

In line with the racial demographics of South-Africa, the number of white South-Africans included in the study was much smaller than the number of black South-Africans included in the study, thereby increasing the risk of a type II statistical error when comparing both populations. Nevertheless, in spite of the small number of white South-Africans included, ^68^Ga-PSMA-11 PET/CT uptake by prostate carcinoma in black South-African patients proved statistically significantly higher when compared to white patients for a comparable Gleason score distribution; when normalized for Gleason score, median uptake of ^68^Ga-PSMA-11 PET/CT proved 2.5 times higher in black when compared to white South-Africans.

## Conclusion

^68^Ga-PSMA-11 PET/CT uptake by prostate carcinoma proved significantly higher in Black South-Africans when compared to White South-Africans, especially when normalized for Gleason score, explaining why 98% of the primary prostate carcinoma where readily identified on ^68^Ga-PSMA-11 PET/CT images, including patients with Gleason score 6 tumors. Accordingly, when confirmed by other studies, ^68^Ga-PSMA-11 PET/CT imaging may prove of great clinical value for diagnosis and staging in black patients**.** Also, ^68^Ga-PSMA-11 uptake by primary prostate carcinoma showed significant correlation with serum PSA values, which proved significantly higher in Black South-Africans when compared with white South-Africans at the time of initial diagnosis in line with the well-documented more aggressive behavior of prostate carcinoma in black men versus white men.

## References

[1] Greenlee R, Murray T, Bolden S, Wingo P (2000). Cancer Statistics. CA Cancer J Clin.

[2] Evans S, Metcalfe C, Ibrahim F, Persad R, Ben-Shlomo Y (2008). Investigating black-white differences in prostate cancer prognosis: a systematic review and meta-analysis. Int J Cancer.

[3] Powell I (2011). The precise role of ethnicity and family history on aggressive prostate cancer: a review analysis. Arch Esp Urol.

[4] Mahal B, Aizer A, Ziehr D, Hyatt AS, Lago-Hernandez C, Choueiri TK (2014). Racial disparities in prostate cancer-specific mortality in men with low-risk prostate cancer. Clin Genitourin Cancer.

[5] Yamaoh K, Deville C, Vapiwala N, Spangler E, Zeigler-Johnson CM, Malkowicz B (2015). African American men with low-grade prostate cancer have increased disease recurrence after prostatectomy compared with Caucasian men. Urol Oncol.

[6] Afshar-Oromieh A, Malcher A, Eder M, Eisenhut M, Linhart HG, Hadaschik BA (2013). PET imaging with a [68Ga]gallium-labelled PSAM ligand for the diagnosis of prostate cancer: biodsitribution in humans and first evaluation of tumour lesions. Eur J Nucl Med Mol Imaging.

[7] Afshar-Oromieh A, Zechmann CM, Malcher A, Eder M, Eisenhut M, Linhart HG (2014). Comparison of PET imaging with a (68)Ga-labelled PSMA ligand and (18)F-choline-based PET/CT for the diagnosis of recurrent prostate cancer. Eur J Nucl Med Mol Imaging.

[8] Afshar-Oromieh A, Avtzi E, Giesel FL, Holland-Letz T, Linhart HG, Eder M (2015). The diagnostic value of PET/CT imaging with the (68)Ga-labelled PSMA ligand HBED-CC in the diagnosis of recurrent prostate cancer. Eur J Nucl Med Mol Imaging.

[9] Eiber M, Maurer T, Souvatzoglou M, Beer AJ, Ruffani A, Haller B (2015). Evaluation of hybrid 68Ga-PSMA ligand PET/CT in 248 patients with biochemical recurrence after radical prostatectomy. J Nucl Med.

[10] Afshar-Oromieh A, Holland-Letz T, Giesel FL, Kratochwil C, Mier W, Haufe S (2017). Diagnostic performance of 68Ga-PSMA-II (HBED-CC) PET/CT in patients with recurrent prostate cancer: evaluation in 1007 patients. Eur J Nucl Med Mol Imaging.

[11] Eder M, Schäfer M, Bauder-Wüst U, Hull WE, Wangler C, Mier W (2012). 68Ga-complex lipophilicity and the targeting property of a urea-based PSMA inhibitor for PET imaging. Bio Conjug Chem.

[12] Zechmann CM, Afshar-Oromieh A, Armor T, Stubbs JB, Mier W, Hadaschik B (2014). Radiation dosimetry and first therapy results with a (124)I/(131)I-labeled small molecule (MIP-1095) targeting PSMA for prostate cancer therapy. Eur J Nucl Med Mol Imaging.

[13] Ebenhan T, Vorster M, Marjanovic-Painter B, Wagener J, Suthiram J, Modiselle M (2015). Development of a single vial kit solution for radiolabeling of 68Ga-DKFZ-PSMA-11 and its performance in prostate cancer patients. Molecules.

[14] Maurer T, Gschwend JE, Rauscher I, Souvatzoglou M, Haller B, Weirich G (2016). Diagnostic efficacy of (68)Gallium-PSMA positron emission tomography compared to conventional imaging for lymph node staging of 130 consecutive patients with intermediate to high risk prostate cancer. J Urol.

[15] Budäus L, Leyh-Bannurah S, Salomon G, Michl U, Heinzer H, Huland H (2016). Initial experience of (68)Ga-PSMA PET/CT imaging in high-risk prostate cancer patients prior to radical prostatectomy. Eur Urol.

[16] Uprimny C, Kroiss A, Decristoforo C, Fritz J, von Guggenberg E, Kendler D (2017). 68Ga-PSMA-11 PET/CT in primary staging of prostate cancer: PSA and Gleason score predict the intensity of tracer accumulation in the primary tumour. Eur J Nucl Med Mol Imaging.

[17] Rajasekaran A, Anilkumar G, Christiansen J (2005). Is prostate specific membrane antigen a multifunctional protein?. Am J Physiol Cell Physiol.

[18] Heston W (1997). Characterization and glutamyl preferring carboxypeptidase function of prostate specific membrane antigen: a novel folate hydrolase. Urology.

[19] Yao V, Parwani A, Maier C, Heston W, Bacich D (2008). Moderate expression of prostate-specific membrane antigen, a tissue differentiation antigen and folate hydrolase, facilitates prostate carcinogenesis. Cancer Res.

[20] Yao V, Berkman C, Choi J, O'Keefe DS, Bacich DJ (2010). Expression of prostate-specific membrane antigen (PSMA), increases cell folate uptake and proliferation and suggests a novel role for PSMA in the uptake of the non-polyglutamated folate, folic acid. Prostate.

[21] Koochekpour S, Majumdar S, Azabdaftari G, Attwood K, Scioneaux R, Subramani D (2012). Serum glutamate levels correlate with Gleason score and glutamate blockade decreases proliferation, migration and invasion and induces apoptosis in prostate cancer cells. Clin Cancer Res.

[22] Lapidus R, Tiffany C, Isaacs J, Slusher BS (2000). Prostate-specific membrane antigen (PSMA) enzyme activity is elevated in prostate cancer cells. Prostate.

[23] Conway R, Petrovic N, Li Z, Heston W, Wu D, Shapiro L (2006). Prostate-specific membrane antigen regulates angiogenesis by modulating integrin signal transduction. Mol Cell Biol.

[24] Rajasekaran S, Christiansen J, Schmid I, Oshima E, Ryazantsev S, Sakamoto K (2008). Prostate-specific membrane antigen associates with anaphase-promoting complex and induces chromosomal instability. Mol Cancer Ther.

[25] Tsui P, Rubenstein M, Guinan P (2005). Correlation between PSMA and VEGF expression as markers for LNCaP tumor angiogenesis. J Biomed Biotechnol.

[26] Perico M, Grasso S, Brunelli M, Martignoni G, Munari E, Moiso E, et al. Prostate-specific membrane antigen (PSMA) assembles a macromolecular complex regulating growth and survival of prostate cancer cells “in vitro” and correlating with progression “in vivo”. Oncotarget 2016; 7(45): 74189-74202. 10.18632/oncotarget.12404 PMC5342045 27713116

[27] Shuch B, Mikhail M, Satagopan J, Lee P, Yee H, Chang C (2004). Racial disparity of epidermal growth factor receptor expression in prostate cancer. J Clin Oncol.

[28] Sachpekidis C, Kopka K, Eder M, Hadaschik BA, Freitag MT, Pan L (2016). 68Ga-PSMA-11 Dynamic PET/CT Imaging in Primary Prostate Cancer. Clin Nucl Med.

[29] Kabalin J, McNeal J, Johnstone I, Stamey T (1995). Serum prostate-specific antigen and the biologic progression of prostate cancer. Urology.

[30] Pierozazio P, Desai M, McCann T, Benson M, McKierann J (2009). The relationship between preoperative prostate-specific antigen and biopsy Gleason sum in men undergoing radical retropubic prostatectomy: a novel assessment of traditional predictors of outcome. BJU Int.

[31] Koochekpour S, Buckles E, Shourideh M, Hu S, Chandra D, Zabaleta J (2014). Androgen receptor mutations and polymorphisms in African American prostate cancer. Int J Biol Sci.

[32] Mavropoulos J, Partin A, Amling C, Terris MK, Kane CJ, Aronson WJ (2007). Do racial differences in prostate size explain higher serum PSA concentrations among black men?. Urology.

[33] Moul J, Connelly R, Mooneyhan R, Zhang W, Sesterhenn IA, Mostofi FK (1999). Racial differences in tumor volume and prostate specific antigen among radical prostatectomy patients. J Urol.

[34] Vollmer R (2004). Race and the linkage between serum prostate-specific antigen and prostate cancer: a study of American veterans. Am J Clin Pathol.

